# Supplementary figures and images for: Whole-genome comparison of two same-genotype macrolide-resistant Bordetella pertussis isolates collected in Japan
Source: PLoS One. 2024 Feb 15;19(2):e0298147. doi: 10.1371/journal.pone.0298147 (PMC10868825; doi:10.1371/journal.pone.0298147)

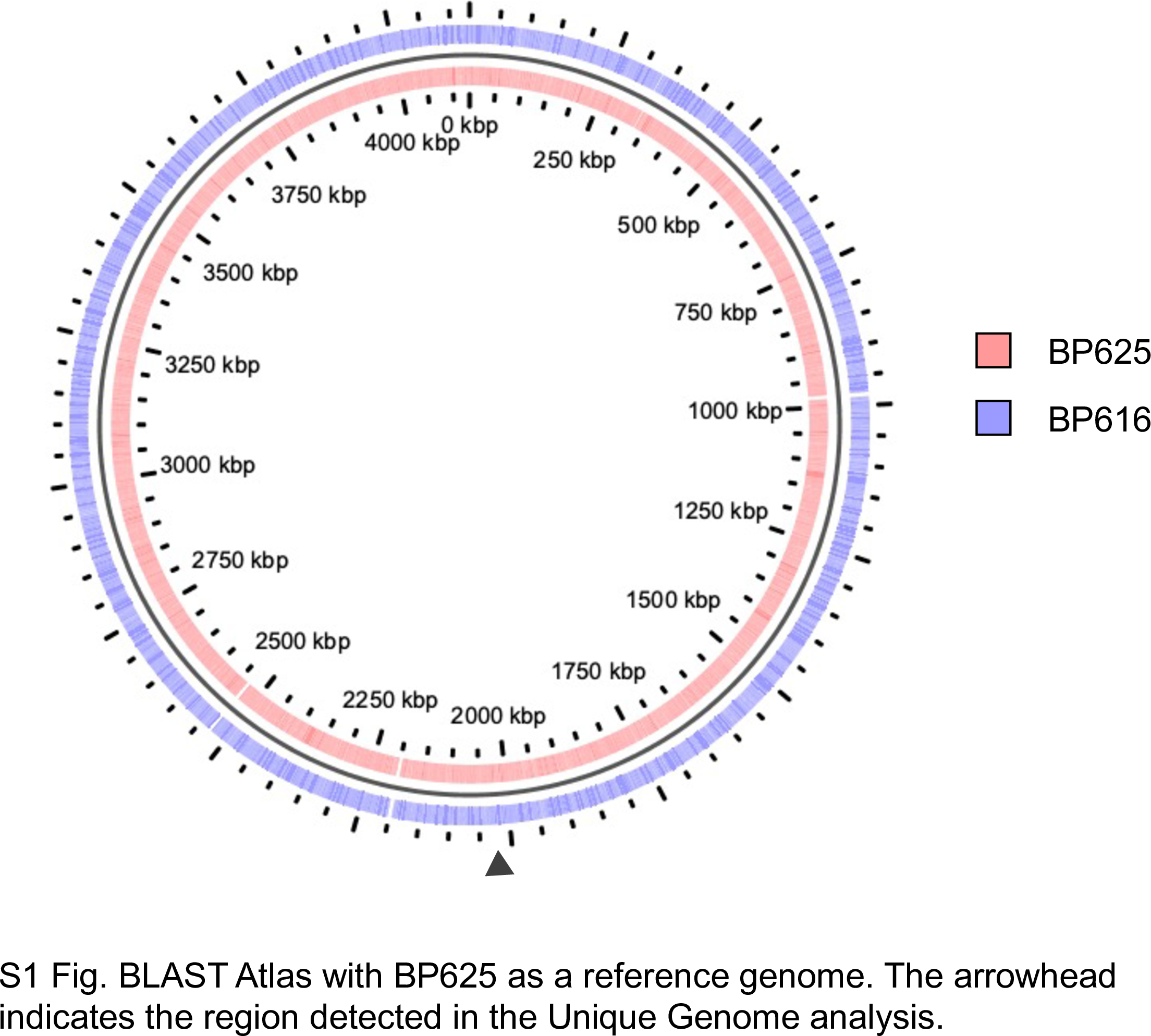

Supplement: S1 Fig — The arrowhead indicates the region detected in the Unique Genome analysis. (TIF) [file pone.0298147.s001.tif]

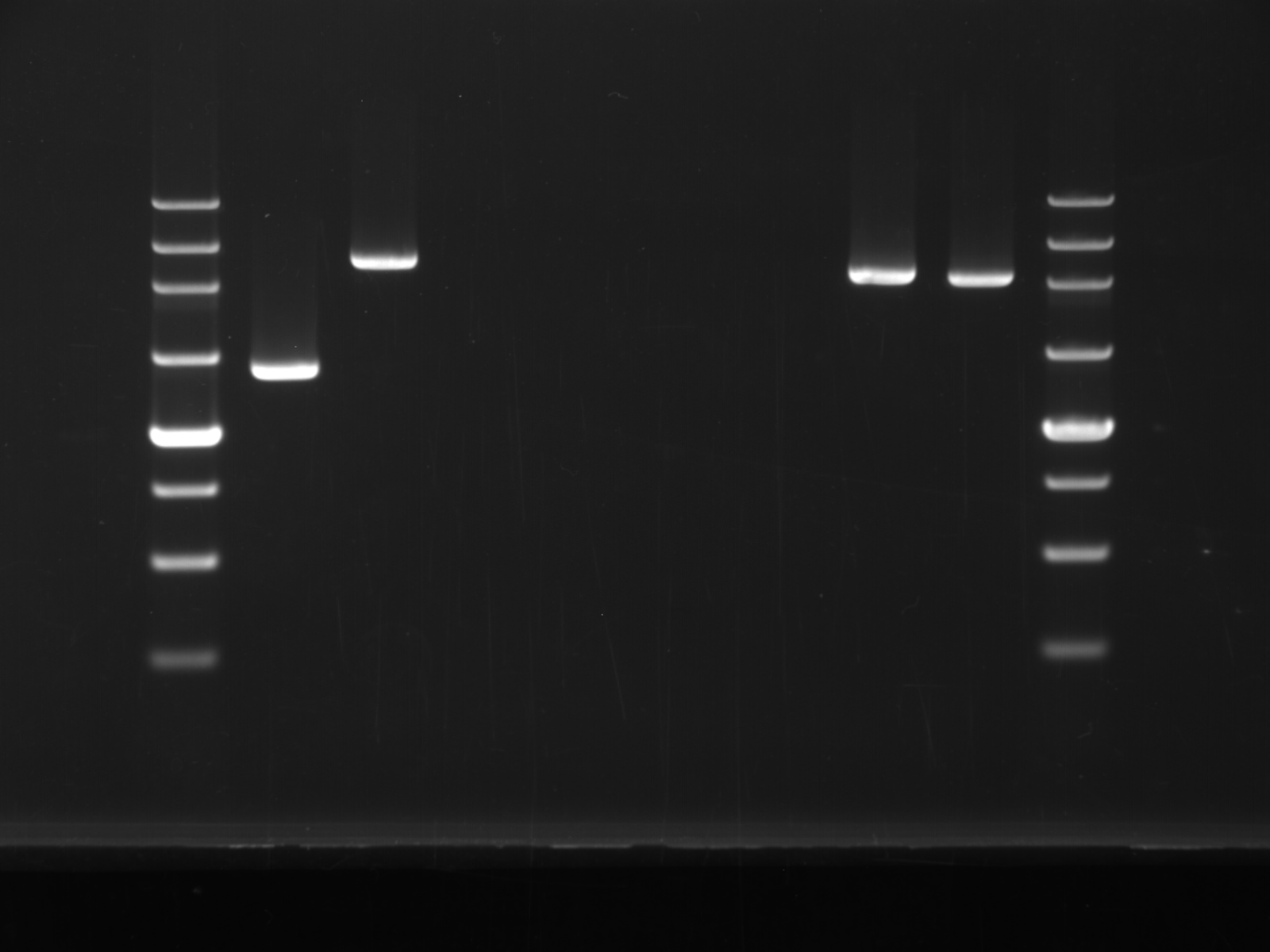

Supplement: S1 Raw images — (ZIP) [file pone.0298147.s003.zip › S1_raw_images_labeled.tif]
